# Current Trends in Aerogel Use in Heritage Buildings: Case Studies from the Aerogel Architecture Award 2021

**DOI:** 10.3390/gels9100814

**Published:** 2023-10-13

**Authors:** Michal Ganobjak, Samuel Brunner, Jörg Hofmann, Verena Klar, Michael Ledermann, Volker Herzog, Beat Kämpfen, Ralf Kilian, Manfred Wehdorn, Jannis Wernery

**Affiliations:** 1 Laboratory for Building Energy Materials and Components, Empa, Swiss Federal Laboratory for Science and Technology, Überlandstrasse 129, 8600 Dübendorf, Switzerland; samuel.brunner@empa.ch; 2 Faculty of Architecture, Institute of History and Theory of Architecture and Monument Restoration, Slovak University of Technology in Bratislava, Námestie Slobody 19, 812 45 Bratislava, Slovakia; 3 WPB Planungsgesellschaft mbH & Co KG, Meyerstraße 56, 99423 Weimar, Germany; hofmann@plangruppe-weimar.de; 4 Klar Architektur + Energieberatung, Bahnhofstr. 19/21, 72127 Mähringen, Germany; mail@klar-architektur.de; 5 Architekturbüro Ledermann AG, Mittelstrasse 40, 4900 Langenthal, Switzerland; m.ledermann@arch-ledermann.ch; 6 Herzog Architektur, Frühlingstrasse 69, 85354 Freising, Germany; volker.herzog@herzog-architektur.de; 7 Kämpfen Zinke + Partner AG, Gutstrasse 73, 8055 Zürich, Switzerland; beat@kaempfen.com; 8 Fraunhofer-Institut für Bauphysik IBP, Fraunhoferstr. 10, 83626 Valley, Germany; ralf.kilian@ibp.fraunhofer.de; 9 Institute for History of Art, Building Archaeology and Restoration, Faculty of Architecture and Planning, Vienna University of Technology, Karlsplatz 13, 1040 Vienna, Austria; manfred@wehdorn.at

**Keywords:** aerogel, architecture, thermal insulation, heritage, retrofit, energy efficiency, aerogel render

## Abstract

Silica aerogels are high-performance thermal insulation materials that can be used to provide unique solutions in the envelopes of buildings when space is limited. They are most often applied in historic buildings due to thin insulation thicknesses and since they are compatible with historic structures. In 2021, the first Aerogel Architecture Award was held at Empa in Switzerland in order to collect, evaluate and award outstanding uses of this relatively new building material. From the submitted projects, three were selected for an award by an expert jury. They showcased applications in which heritage protection and the conservation of a building’s character and expression were reconciled with significant improvements in the energy efficiency of the building. The submissions also showed that a broader communication of these types of solutions is important in order to provide more information and security to planners and heritage offices and to facilitate the application of these materials in the future so that they can contribute to the protection of cultural heritage and reductions in the operational and embodied emissions of our building stock by extending the life expectancy and energy efficiency of existing buildings.

## 1. Introduction

Silica-aerogel-based thermal insulation materials are characterised by their outstanding insulation performance [1] but also by their comparatively high cost. Most of them are superinsulators, i.e., materials with a thermal conductivity of 20 mW/(m∙K) or less, due to their unique micro- and mesoporous structures. These materials consist of a three-dimensional network of silica, forming open pores in the nanometre range, which is covered with hydrophobic surface groups, minimising water uptake. In general, the thermal properties of porous solids are determined by solid conduction, gas conduction, radiation and convection [2]. The latter can be neglected for pores of less than a few millimetres and typically, thermal conductivity is dominated by the conduction of the pore-filling gas. In aerogels, the pore sizes are so small that the transfer of heat in the gas is strongly suppressed by collisions of the gas molecules with the pore walls. This is called the Knudsen effect [1,3]. Hence, in silica aerogels, the main heat transport mechanism is solid conduction (phonons), with radiative heat transfer only becoming dominant at higher temperatures, i.e., above 200 °C. At room temperature, the thermal conductivities of silica aerogel materials are roughly between 13 and 19 mW/(m∙K) [1,4]. In addition to their thermal properties, silica aerogels are typically water vapour diffusion open but hydrophobic at the same time; they can be transparent or translucent [5], and materials with good fire protection (i.e., non-flammable materials) are available [4]. Because of the small thicknesses that are needed to achieve good insulation performances, aerogel insulation provides a technical advantage whenever space is limited or expensive. This can be the case in inner-city projects for new buildings or upward extensions in order to maximise the generated use area (and hence the property value), but also for retrofits and architectural details [6]. However, due to their previously mentioned favourable building physical properties, and since they are easily distinguishable from historically used materials, they represent a very attractive insulation material for energy retrofits of heritage buildings. This is because with relatively thin layers of typically 30–50 mm, they can significantly improve the energy use of such buildings without negatively impacting their appearance. Other common insulation materials such as mineral wool or polymer foams require much thicker layers to achieve the same performance—typically around twice as much—which, in most cases, is not compatible with the requirements of heritage protection. Hence, the superinsulating quality of aerogel materials is a unique feature for their application in historic buildings. This is in spite of the fact that by volume, silica aerogels are much more expensive than conventional materials by up to a factor of ten or more. However, when considering the same insulation performance, including the installation of materials, the cost is of the same order of magnitude, varying by a factor of typically less than three. Also, many conventional insulation materials, e.g., mineral wool or biomass-based materials, take up more moisture, which can be problematic; on the other hand, some, such as expanded polystyrene, are water vapour closed. The latter poses significant risks for condensation and moisture damage in historic buildings and is thus not suitable in most cases.

Detailed descriptions of the early applications of silica aerogels can be found in [7,8,9]-with the first larger project realised in 2008-and their use in buildings is elaborated in [3,10]. Typical materials are blankets, usually 10 mm thick, with a thermal conductivity of around 15–18 mW/(m∙K), boards of variable thicknesses and a thermal conductivity of 17–19 mW/(m∙K), and renders with a thermal conductivity of 27 mW/(m∙K) or more. A more detailed overview of these common silica-aerogel-based materials and products for application in heritage buildings, as well as examples of their use, can be found in Ganobjak et al. [4]. Blowing in cut-offs from aerogel blankets was demonstrated as a new approach to filling cavity walls [11]. Silica aerogel insulating renders were first described by Stahl and colleagues [12] and have been studied in terms of their technical properties and applications [13], durability [14,15] with respect to the addition of fibres [16] and their environmental impact [17]. Also, their moisture behaviour was studied under different wetting conditions, and they showed minimal moisture uptake [18]. In façades which are highly exposed to wind-driven rain, relative humidity could increase in the render, resulting in a concomitant increase in thermal conductivity of up to 9%. However, more research is still needed in order to better describe the hygrothermal, mechanical and durability characteristics of silica aerogel renders [19]. The durability of silica aerogels by themselves in the forms of granules and blankets was studied via accelerated aging under high levels of moisture and solar radiation. The authors found that the thermal performance of these materials did suffer under extreme conditions with an increase of about 10%; however, in common building applications, such “aging” would take a very long time [20]. Due to the higher cost and higher embodied emissions of silica aerogel insulation compared to conventional materials, it is useful to consider the economic and environmental payback times for these solutions [6,21,22,23].

A more recent new use of silica aerogels is the creation of structured façades in historic buildings using silica aerogel renders [24,25]. This innovative approach demonstrates the versatility of such renders and their potential for reducing the energy demand of historic buildings without impacting their appearance if the existing render is not under heritage protection. Another new development is an aerogel-granule-based slurry that is injected into cavity walls where it hardens and creates highly competitive cavity insulation [26].

Even though silica aerogel insulation materials have been an active area of research for several decades and the first projects were realised over 15 years ago, there is still a lack of knowledge about the performance and behaviour of these materials amongst building professionals and heritage experts. This hinders the effective application of aerogel insulation and makes the process of designing with these materials more challenging.

The Aerogel Architecture Award was launched in 2021 by Empa’s Laboratory for Building Energy Materials and Components [27]. The goal was to give more visibility to successful applications of silica aerogels and to make knowledge of the potential of these materials—for both retrofits and new buildings—more accessible. Three projects were awarded that showcased the successful and creative use of silica aerogels in heritage buildings in order to enhance the buildings’ energy efficiency and resilience which, in turn, increased their sustainability and protected their cultural heritage. We present these projects here in order to expand the knowledge on the use of silica aerogel in buildings, especially cultural heritage buildings. The practical examples detail the use of aerogels and explain the reasoning for the material choice.

## 2. Realised Aerogel Projects

The five projects submitted from the countries Switzerland, Germany and Austria are summarised in Table 1, and details can be found in [27]. The projects were rated by an expert jury of four members—Volker Herzog, Beat Kämpfen, Ralf Kilian and Manfred Wehdorn—all of whom are architects and/or experts on monument conservation. The judgement criteria for the submitted projects were as follows:

i. The preservation of appearance in the case of cultural heritage;
ii. The energy efficiency of the concept;
iii. The originality of the solution.

The description in this article attempts to follow this structure. The jury selected three projects to be awarded in a ceremony at Empa in Dübendorf, Switzerland, which are described in more detail in the following sections. The winning projects all used silica aerogel renders, and they were all located in the CfB Oceanic climate zone according to the Köppen climate diagram. Hence, they had similar requirements regarding climate conditions. Also, since they were all historic buildings under heritage protection, in the design process, it was necessary to determine what the tangible value holders were. In general, these are defined by the specific building’s local monument board as worthy of preservation. In the design process, it is then up to discussion whether and in which position aerogel materials can be used.

### 2.1. Bauhaus-Universität Weimar

The building of the F. A. Finger-Institute for Building Material Science of the Bauhaus University is located centrally in the inner city of Weimar and is recognised as an outstanding example of Socialist Modernism as a single monument. It houses laboratory, examination, office and teaching rooms. Because of the poor state of the original exterior render, the task at hand was the extensive refurbishment of the façade, including the wooden and aluminium façade window elements.

**Figure 1:**
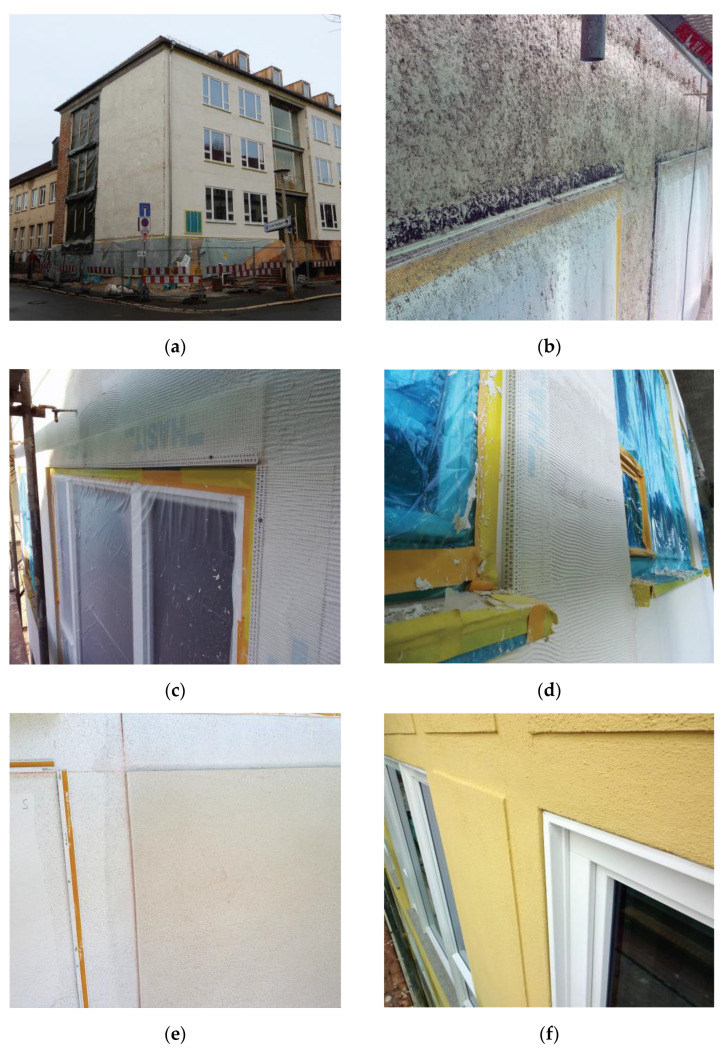
Some details of the Bauhaus-Universität during the retrofitting process: (**a**,**b**) before renovation, (**c**–**e**) during renovation (**f**) and final details. Images: WPB Planungsgesellschaft mbH & Co KG.

It was possible to install thermally insulating windows approximately within the measures of the original profile. The energetic retrofit of the rendered areas, which was required as well due to the high thermal losses, seemed impossible due to the non-negotiable requirement of the Thuringian State Office for the Preservation of Monuments and Archaeology to produce a render surface at the same level as the connections of the travertine plinth cladding and the profile of the cast stone eaves, in accordance with the original state (Figure 1a). The option of implementing interior thermal insulation at a later time was also out of the question because of the high-quality equipment involved, including scanning electron microscopes. It was only through intensive research into the technical status of alternative building materials, including vacuum insulation systems, and the practical possibilities of their use that the possibility of using high-performance thermal insulation renders crystallised.

After detailed planning and energy calculations, it became clear that the use of an aerogel render from HASIT (a sister company of Fixit) would provide a solution that would not only significantly improve the existing U-value from 1.36 W/(m²∙K) to 0.58 W/(m²∙K) and thus significantly reduce the building’s energy loss, optimise the dew point situation and all component connections in terms of energy, but would also meet the requirements for the preservation of historical monuments. With the construction-tested render structure, from the levelling render to the colour coating on a purely mineral basis, it was also possible to meet the fire protection requirements of an A1 thermal insulation system—which is non-combustible—and at the same time maintain the constructional material quality of a purely mineral render façade, the main advantages of which is good diffusion openness and moisture-regulating properties.

The realised render structure was thoroughly tested in advance by employees of the F.A. Finger Institute and inspected during construction. Intensive technical support and advice from the HASIT staff, including a special colour formulation, enabled both the plastering company and the architect to make technically sound decisions at any time.

**Figure 2:**
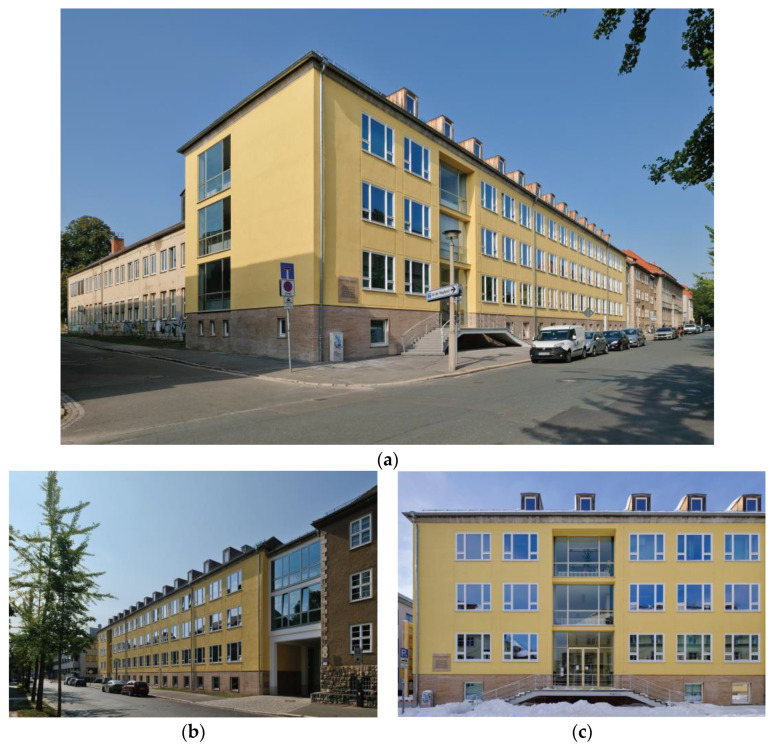
(**a**–**c**) Impressions of the Bauhaus-Universität after its retrofit with the aerogel render. Images: Michael Miltzow—Bildwerk.

**Figure 3:**
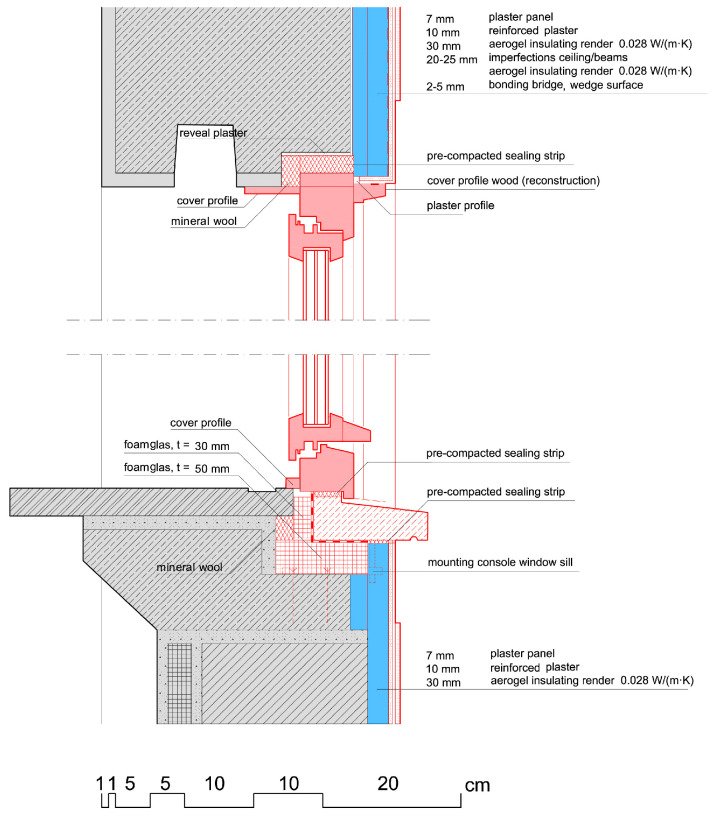
Drawings of details of the retrofit of the Bauhaus-Universität. Drawing: WPB Planungsgesellschaft mbH & Co KG.

With the application of trowel-rendered surfaces on the 30 mm thick, high-performance thermal insulation render FIXIT 222, the historical appearance of the façade could be faithfully restored over the strictly geometrically divided render cassette surfaces and render bands, and thus the overall appearance could meet the requirements of the single monument and a contemporary energetic façade renovation (Figure 2). A detail of the retrofit is given in Figure 3.

### 2.2. Neckarhalde Tübingen

**Figure 4:**
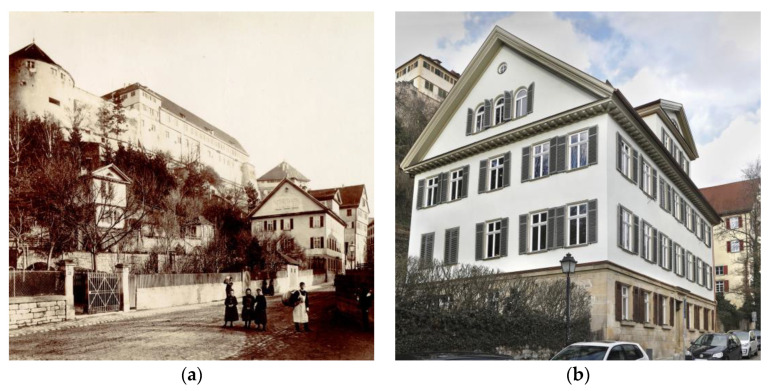
Residential building of Neckarhalde 32 in a very early picture from around 1900 (on the right-hand side of the picture), with Hohentübingen Castle in the background, (**a**) and after its retrofit with aerogel in 2017–2018 (**b**). Images: Stadtarchiv Tübingen and Anne Faden.

The stately residential building at Neckarhalde 32 in Tübingen was built below the castle in 1830, with a ground floor in sandstone and two rendered upper floors in a half-timbered construction (Figure 4a).

The broad house is characteristic of the first residential buildings that were built in the early 19th century directly in front of the ramparts of the old cities in open construction, mostly on new and attractive streets and in front of a large garden plot. In terms of design, its main impact is the axially symmetrical structure, with its central entrance reaching up to the belt cornice, the dominant roof house on the street façade and the fitting of all windows with folding shutters.

Reading the justification for its monument status and looking at the historic plans and photographs, it is easy to see that the façade of the residential building has survived largely unchanged. Since, in addition to the protected façade, the interior is characterized by features such as period lambrequins and profiled wooden window reveals, the question arose as to how thermal insulation could be achieved with the slimmest possible structure.

**Figure 5:**
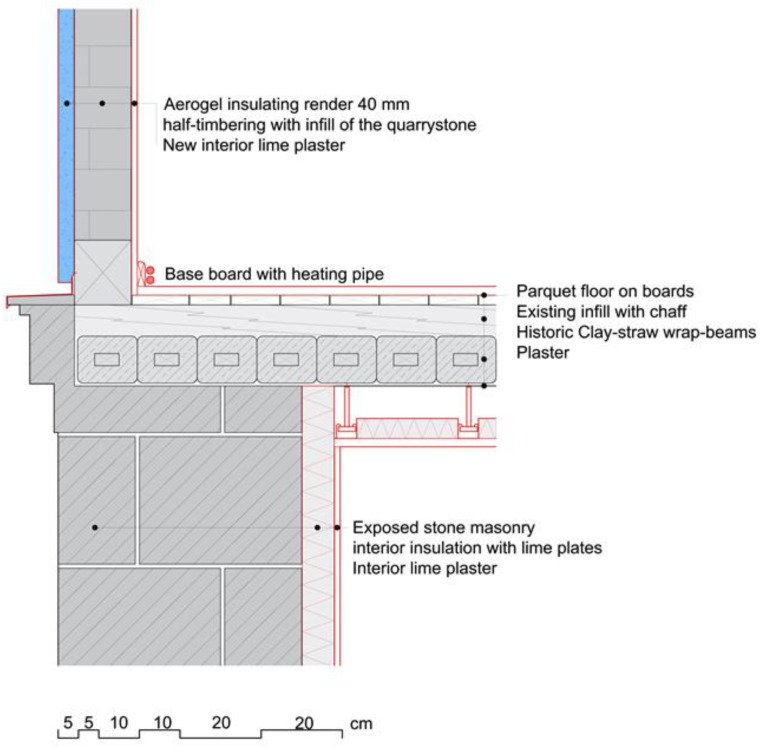
Vertical section of the transition from the ground floor to the first floor. Drawing: adjusted from Gerhard Schmid.

The solution was a state-of-the-art aerogel insulating render whose positive properties motivated those involved in the project to use it for the first time on a monument in the region: first and foremost was its very good insulating effect with a low application thickness, its high diffusion openness, its good workability and its composition, which consisted of lime as a binder and silicate aerogel as an insulating material. The initially sceptical monument authorities were convinced of its use on the basis of built examples from Switzerland. Approximately 4 cm of aerogel render was sprayed onto an undulating render base on the main façades (Figure 5) and followed by a fine-grained, hand-brushed finishing render.

A restorer uncovered historic paint finishes, which formed the basis for the final coat of silicate paint with a light, greenish tint (Figure 4b). Together with further very comprehensive energy-saving measures, the building achieved the KfW standard “Effizienzhaus Denkmal”, a German retrofit standard for historic buildings that requires a building to have at most an energy need of 160% of a comparable reference building which adheres to the German energy regulations [28]. The building fulfils these energy-saving regulations for renovations without losing its stylish appearance and its significance as a testimony to its time. Thanks to the heating supplied by a pellet boiler, the historically valuable building also has a very good CO_2_ balance.

### 2.3. Mühlestock Madiswil

The presented building conversion realisation concerns the former mill in Madiswil in Switzerland. The building, which dates back to 1844, has been classified as worthy of protection by the cantonal office for the preservation of historical monuments. The owner wished to expand the living space to the ground floor, which was originally used as the mill room. The challenge was to realise a pleasant and respectable implementation for living, dining and cooking with the old masonry. The second and third floors now house bedrooms and bathrooms. The ground floor has always remained a “great hall” where the functions of the living room, and kitchen and dining room come together without spatial separation, maintaining the industrial character of the original use.

**Figure 6:**
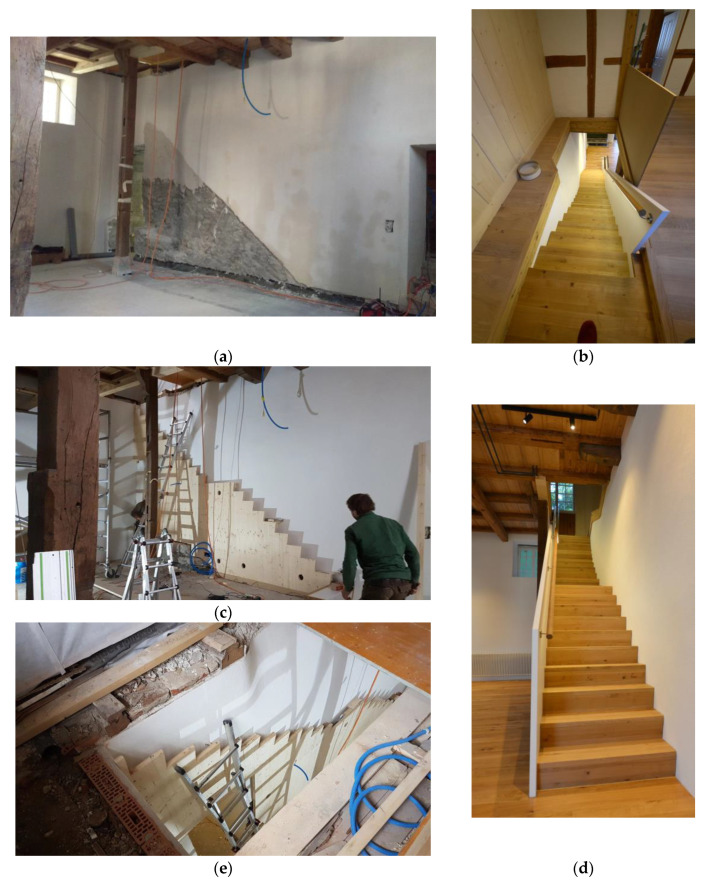
Impressions of the process of retrofitting the wall behind the staircase and building the wall (**a**,**c**,**e**), as well as the finished staircase viewed from top and bottom (**b**,**d**). Images: Architekturbüro Ledermann AG (Michael Ledermann).

**Figure 7:**
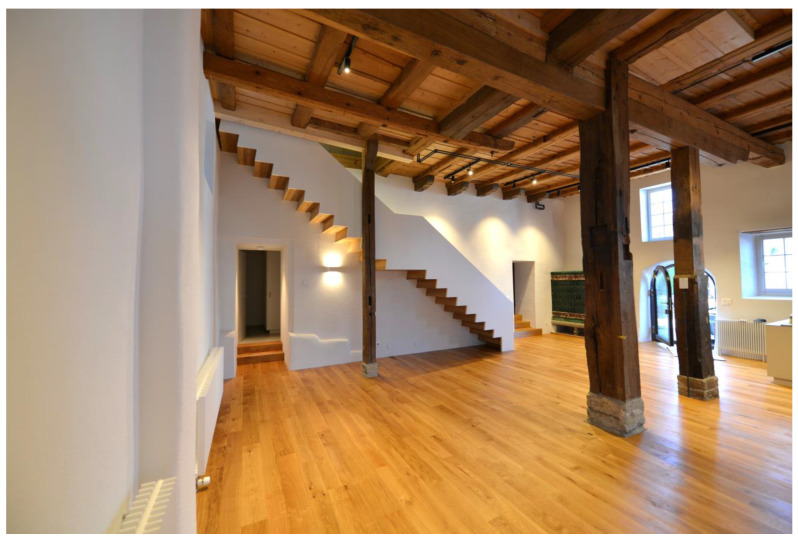
The “great hall” after the completion of the retrofit of the wall under the staircase. Image: Architekturbüro Ledermann AG (Michael Ledermann).

**Figure 8:**
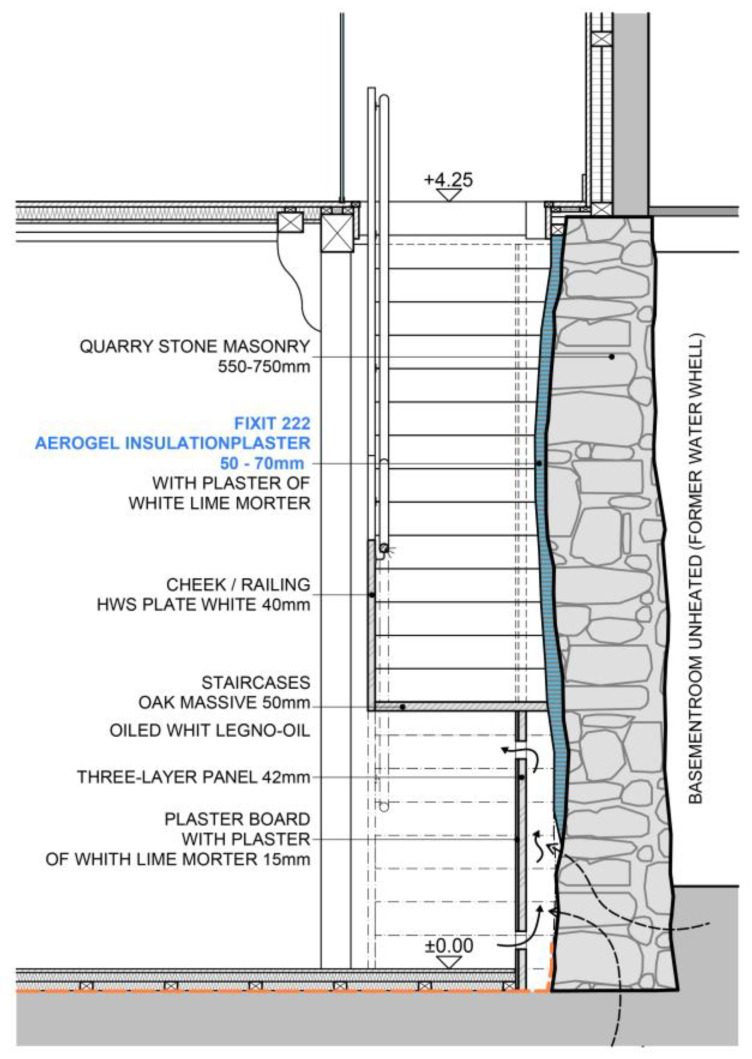
Technical drawing of the wall retrofit with staircase. Drawing: Architekturbüro Ledermann AG.

The exterior walls of the ground floor are made of 50–70 cm quarry stone masonry. To preserve their external appearance, we chose the aerogel Fixit 222 interior insulating render. The presented detail concerns the 50 cm thick partition wall, which is also made from quarry stone, between the “great hall” and the former engine room, where the waterwheel and the machinery were located. The stairs to the second floor were placed along this wall. The quarry stone wall had many irregularities, and due to its composition, it was not possible to affix the new stairs to it. Instead of creating a second faux wall, it was decided that the irregularities in the existing quarry wall should be emphasised. A back ventilated supporting structure, which holds the stairs from below, was installed in front of the wall under the steps of the stairs. This solution allowed for ventilation in the lower area of the wall in order to avoid the possible build-up of humidity. The visible area of the wall above the stairs was insulated with the aerogel insulating render to maintain the irregularities of the wall and to preserve the original characteristic of the room. The retrofitting process is shown in Figure 6a,c,e, the result in Figure 6b,d and the "great hall" after the retrofit in Figure 7. The staircase solution is detailed in Figure 8.

## 3. Comments of the Jury Members

Based on their individual evaluations and thorough discussions with all members, the jury decided to award two second-place prizes to the projects Bauhaus-Universität Weimar and Neckarhalde Tübingen and the first-place prize to the project Mühlestock Madiswil. The jury comments for the three awarded projects are presented below.

### 3.1. Bauhaus-Universität Weimar—Second Place, Shared

#### 3.1.1. Volker Herzog

“The Bauhaus façade is structured by pillars that subdivide the large glass surfaces. Insulation can only be applied in small thicknesses to preserve this richly detailed façade. The solution with the insulating render meets the design requirements of monument preservation and significantly improves the energy performance of the building”.

#### 3.1.2. Beat Kämpfen

“For operational reasons, the energy renovation had to be limited to the exterior. The functional-modern expression of the laboratory building with the windows arranged almost flush with the façade was preserved by replacing the old, damaged render with aerogel insulation render. The thermal insulation level is consistently guided and thermal bridges could be completely avoided. The project shows a carefully designed façade renovation”.

#### 3.1.3. Ralf Kilian

“The refurbishment of modern architecture is one of the central challenges in the preservation of historical monuments today. This example shows how the use of aerogel renders can help to achieve successful renovations and energy savings in monuments, even with thin walls”.

#### 3.1.4. Manfred Wehdorn

“The building of the F. A. Finger-Institute for Building Material Science of the Bauhaus University is an interesting example for the protection of contemporary architecture. The project demonstrates exemplarily the advantages of the aerogel render for thermal insulation, which can be used in protected buildings too. The professional support of the restauration by an international institute for building materials science, which uses the building too, underlines the importance of the project for aerogel materials”.

### 3.2. Neckarhalde—Second Place, Shared

#### 3.2.1. Volker Herzog

“This project convinces with the insulation of the half-timbered façade and the preservation of the historical substance in a historically significant environment. It is a good example that even half-timbered houses can be operated economically in the future”.

#### 3.2.2. Beat Kämpfen

“The project was conceived and implemented in a holistic, ecological and sustainable way. Materials were continued to be used as much as possible, the interventions were carried out very carefully and coordinated sensibly. Thanks to the replacement of the render with a 4 cm thick aerogel exterior render, the heat loss of the façades could be significantly reduced. The volumetry was preserved and the architectural expression was clarified. The energetic renovation is exemplary”.

#### 3.2.3. Ralf Kilian

“In this project, the overall energetic concept should be emphasised as well as the fine design of the façade in this important urban area”.

#### 3.2.4. Manfred Wehdorn

“The project in Tübingen is a representative example of the renovation of residential buildings from the early 19th century, of which there are many in Europe. In terms of monument preservation, the insulating aerogel render used demonstrates its advantages, such as the excellent insulating effect, the low application thickness, the high diffusion openness and much more. The aerogel product used was thus fundamental to achieving the so-called “KfW—Effizienzhaus—Denkmal”. In addition, the building was the first in the region in which an aerogel product was used in the preservation of a historical monument and convinced both the residents and the responsible decision-makers”.

### 3.3. Mühlestock—First Place

#### 3.3.1. Volker Herzog

“The project is convincing because the energy improvements were also taken into account when renovating the façade in accordance with the preservation order. This is an incentive to also renovate monuments for energy efficiency”.

#### 3.3.2. Beat Kämpfen

“The small renovation shows that with detailed and sensitive planning and the use of new materials, historical building fabric can be respectfully renewed and energetically improved. The aerogel render is excellently suited to make the old building fabric with all its crooked shapes still tangible. Any moisture problems are countered with a very simple and sympathetic measure”.

#### 3.3.3. Ralf Kilian

“This example, which won the first place of the award, shows that high-performance aerogel insulating renders can be used to create uneven surfaces in particular, where conventional board insulation materials reach their limits. What is striking about the project is the staging of the old and the existing, which is literally celebrated here in its value”.

#### 3.3.4. Manfred Wehdorn

“The project of the mill building in Madiswil is a good example of the third-party use of (pre-)industrial buildings—a topic that is still to be regarded as a ‘mainstream’ of contemporary monument conservation. In addition to the thermal qualities of the used aerogel, the project shows how this material is able to absorb the irregularities in the surfaces of historic renders and thus serves to preserve the “age value” that is always cited. In this example, aerogel emphatically demonstrates its importance for the practical preservation of historical monuments”.

## 4. Conclusions and Outlook

The first edition of the Aerogel Architecture Award was held in order to collect and promote exemplary and innovative cases of the use of aerogel superinsulation in buildings. While the call for the award was not limited to protected buildings, all the submitted projects were retrofits of historic buildings, reflecting the most common use of aerogel materials in buildings at the moment. The three awarded projects demonstrated how silica aerogel materials can be used to preserve the character and appearance of historic and protected buildings while at the same time significantly improving their energy efficiency and thermal comfort, e.g., by reducing the U-value by almost 60% in the Bauhaus-Universität project or by reaching the KfW standard for monuments for the Neckarhalde project in Tübingen. An aerogel render was selected as the most suitable solution in all three projects as it allowed for applications on uneven surfaces, the modulation of the façade and the deliberate creation of an uneven, “organic” surface, as demonstrated in the winning project of the mill in Madiswil. The projects also highlight the importance of the communication of successful applications of aerogel insulation in protected buildings since it helps monument offices evaluate and facilitate retrofit proposals using aerogel insulation. The energetic retrofit of a heritage building represents a special case of refurbishment with a primary focus on preserving the building’s historical value. Prioritising the conservation of these structures and materials, along with efforts to reduce energy consumption and maintenance requirements, maintaining stable environmental conditions for the construction (e.g., temperature and humidity), and proper use can substantially prolong their lifespan as valuable artefacts from the past. In future projects, it would be helpful if, in addition to thermal calculations or simulations, in situ measurements of the U-value of the building envelope before and after the retrofit were also performed in order to better quantify the performance improvements as well as to study the long-term behaviour of aerogel materials.

Finally, the collection of submitted examples and their calculated energetic performance values suggest that using aerogel materials in heritage buildings not only leads to significant savings in energy and operational costs [29] but also sets a compelling example for the refurbishment of non-protected buildings. This is crucial in order to increase the retrofitting rates of the existing building stock in Central and Northern Europe [30], one of the most significant measures needed for reducing the operational and embodied emissions of the built environment in this geographical area.

## Figures and Tables

**Table 1:** The five projects submitted to the Aerogel Architecture Award 2021. The rows indicate the location of the project, and a picture after each project’s completion, the location of aerogel application, the thickness and type of the applied material and the size of the area that was insulated with aerogel. All submitted buildings are located in the CfB Oceanic climate zone according to the Köppen climate diagram. Images (l.t.r.): Seraina Wirz and Rolf Schaffner; Trimmel Wall Architekten; Michael Miltzow—Bildwerk; Architekturbüro Ledermann AG (Michael Ledermann); Anne Faden.

Lettenstrasse, Zurich, Switzerland	Mariahilferstrasse, Vienna, Austria	Bauhaus-Universität, Weimar, Germany	Mühlestock, Madiswil, Switzerland	Neckarhalde, Tübingen, Germany
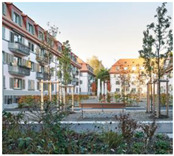	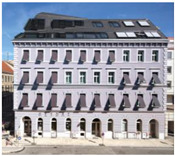	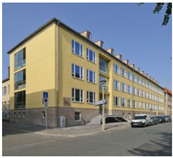	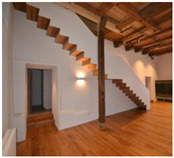	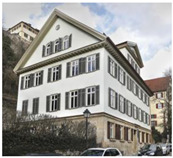
inside insulation	outside insulation	outside insulation	inside insulation	outside insulation
40–60 mm aerogel render; 50 mm aerogel blanket for dormer windows	50 mm aerogel render	30 mm aerogel render	50–70 mm aerogel render	40 mm aerogel render
1260 m^2^ of render; 50 m^2^ of blanket	250 m^2^	537 m^2^	130 m^2^	355 m^2^

## Data Availability

Not applicable.
